# Genotype-Phenotype Correlation of G6PD Mutations among Central Thai Children with G6PD Deficiency

**DOI:** 10.1155/2021/6680925

**Published:** 2021-02-09

**Authors:** Boonchai Boonyawat, Tim Phetthong, Nithipun Suksumek, Chanchai Traivaree

**Affiliations:** ^1^Division of Medical Genetics, Department of Pediatrics, Phramongkutklao Hospital and Phramongkutklao College of Medicine, Bangkok, Thailand; ^2^Division of Neonatology, Department of Pediatrics, Phramongkutklao Hospital and Phramongkutklao College of Medicine, Bangkok, Thailand; ^3^Division of Hematology/Oncology, Department of Pediatrics, Phramongkutklao Hospital and Phramongkutklao College of Medicine, Bangkok, Thailand

## Abstract

**Background:**

Glucose-6-phosphate dehydrogenase (G6PD) deficiency is the most common X-linked inherited erythroenzymopathy in Thailand. The clinical and hematological manifestations of G6PD deficiency are variable.

**Objective:**

This study aimed to characterize the genotype-phenotype correlation of G6PD mutations in Thai pediatric patients who were followed-up in Phramongkutklao Hospital, a tertiary center in central Thailand. *Material and Method*. A total of 102 children including 73 males (71.6%) and 29 females (28.4%) were included in our study. Mutation analysis was performed by direct DNA sequencing of all coding exons of the G6PD gene. Ninety-one patients (89.2%) were presented with neonatal hyperbilirubinemia and 11 patients (10.8%) were presented with acute hemolytic anemia beyond the neonatal period.

**Results:**

Molecular analysis of the G6PD gene in 102 G6PD-deficient Thai children identified 12 different mutations. G6PD Viangchan (871G > A) and G6PD Canton (1376G > T) were the first (46.2%) and the second (15.4%) most common identified mutations among both male and female G6PD-deficient individuals, respectively. All affected males were hemizygous for G6PD mutations and had an average G6PD level of 16.7 ± 11.5 (3–76) IU/ml.RBC. Majority of female patients (27 in 29, 93.1%) were heterozygous for G6PD mutations and had an average G6PD level of 133.6 ± 43.4 (9–195) IU/ml.RBC. Two female patients (6.9%) were either homozygous or compound heterozygous for the mutations and had G6PD level in the affected male range (35 and 10 IU/ml.RBC). Only 1 in 27 heterozygous females (3.7%) had G6PD level in the affected male range (9 IU/ml.RBC) which is possibly explained by nonrandom X-chromosome inactivation. The correlation of genotypes, G6PD levels, and clinical phenotypes was not demonstrated in our study in which all of the included G6PD-deficient patients were presented with neonatal hyperbilirubinemia and acute hemolytic anemia, since the genotype-phenotype correlation is normally demonstrated in chronic nonspherocytic hemolytic anemia (CNSHA) G6PD-deficient individuals.

**Conclusion:**

This study characterizes the molecular heterogeneity of G6PD variants causing G6PD deficiency in Thai children. Our study demonstrated the efficiency of direct DNA sequencing which can identify 12 missense mutations in Thai children.

## 1. Introduction

Glucose-6-phosphate dehydrogenase (G6PD) deficiency (OMIM 300908) is the most common X-linked inherited erythroenzymopathy in Thailand. The prevalence of G6PD deficiency in Thai populations was 3 to 18% [1]. G6PD deficiency is caused by mutation in the G6PD gene (OMIM 305900), located on the long arm of the X chromosome (Xq28). The G6PD gene consists of 13 exons and encodes a 515-amino acid G6PD enzyme (OMIM 305900). Mutation of the G6PD gene results in diminished functionality or stability of the enzyme, giving rise to a wide range of biochemical heterogeneity and clinical manifestations. To date, more than 180 different mutations in the G6PD gene have been reported in the literature [2, 3]. The majority of the mutations are single-base substitutions leading to missense variants. Many of these mutations occur at relatively high frequencies within specific populations, geographic regions, and ethnic groups [4].

The clinical and hematologic manifestations of G6PD deficiency vary; in most cases, neonatal hyperbilirubinemia and acute hemolytic anemia precipitated by exogenous oxidative stress such as infections, drugs, and fava beans (favism) are principle clinical presentations. In Thailand, most children affected with G6PD deficiency presented with neonatal hyperbilirubinemia [5]. As for X-linked inheritance, symptomatic patients are mostly hemizygous affected males but less common homozygous/compound heterozygous females. Heterozygous females can have a wide range of G6PD activity ranging from normal to severe deficiency due to skewed X-chromosome inactivation. Thus, heterozygous females are also at increased risk of neonatal hyperbilirubinemia and acute hemolysis [3, 5]. Comprehensive analysis of the G6PD mutation and genotype-phenotype correlation of G6PD deficiency in Thai populations have never been studied.

In this study, the genotyping of the G6PD gene and the genotype-phenotype correlation among 102 pediatric patients affected with G6PD deficiency was characterized in Phramongkutklao Hospital, a tertiary care center in central Thailand.

## 2. Material and Methods

### 2.1. Patient Selection

To determine the molecular characterization of the G6PD gene, 102 pediatric patients with G6PD deficiency including 73 males (71.6%) and 29 females (28.4%) who were admitted to Pediatrics Department, Phramongkutklao Hospital, between June 2017 and May 2018 were enrolled in our study. The ages ranged from 1 day to 13 years. Clinical manifestations of our patients included neonatal hyperbilirubinemia and acute hemolysis related to oxidative stress. Chronic nonspherocytic hemolytic anemia associated with G6PD deficiency was not identified in this study. The study protocol was approved by the Institutional Review Board of Royal Thai Army, Thailand. The consent was obtained by the study participants prior to study commencement.

### 2.2. Mutation Analysis of the G6PD Gene

After informed consent was obtained from the patients or parents, a total of 102 EDTA blood samples from all individuals were collected. Genomic DNA was extracted from peripheral blood lymphocytes according to the manufacturer's instructions. All 13 coding exons and exon-intron boundaries of the G6PD gene were amplified by PCR using primers as previously described [6]. The PCR condition was initial denaturing at 95°C for 5 min, followed by denaturing at 95°C for 30 sec, annealing at 62–72°C for 60 sec, extension at 72°C for 60 sec for 30 cycles and final extension at 72°C for 5 min. Final 50 *µ*L PCR reaction mixture contained 100–200 ng genomic DNA, 1X PCR buffer, 1.5 mM MgCl_2_, 200 *µ*M dNTPs, 0.25 *µ*M of each primer, and 0.2 unit Taq DNA polymerase (Thermo Scientific, CA, USA). The PCR products were visualized on 1% agarose gel electrophoresis. All PCR products were purified and directly sequenced in both directions. The reference sequences were NM_001360016.1 and NP_001346945.1 for G6PD cDNA and G6PD amino acid positions, respectively.

### 2.3. G6PD Quantitative Enzymatic Activity

The activity of G6PD enzyme was performed by the enzyme kinetic method (Modified Calbiochem) subsequent to hospital admission and repeated at the steady state approximately 3 months after neonatal jaundice and acute hemolysis. The G6PD enzymatic activity was expressed as international units (IU)/ml.RBC. The normal G6PD enzymatic activity in our laboratory for both males and females was 159–297 and 197–331 IU/ml.RBC, respectively. The G6PD enzymatic activity below the normal range was considered as G6PD deficiency.

### 2.4. Statistical Analysis

Baseline values of selected variables were calculated as mean, standard deviation, and range. Continuous variables were compared between the two groups using the unpaired *t*-test for data with a parametric distribution. Statistical analysis was performed using IBM SPSS Software, Version 23 (IBM, NY, USA), and *p* value <0.05 was considered to be statistically significant.

## 3. Results

A total of 102 children including 73 males (71.6%) and 29 females (28.4%) were included in our study. Clinical manifestations of our G6PD-deficient children were classified as 91 individuals (89.2%) with neonatal hyperbilirubinemia (66 males and 25 females) and 11 individuals (10.8%) with acute hemolytic anemia (7 males and 4 females) (Table 1). The majority (80 patients, 78.4%) of our patients were residing in central Thailand. The remaining patients lived in northeastern (17 patients, 16.7%), northern (4 patients, 3.9%), and southern (1 patient, 1%) Thailand.

**Figure 1:**
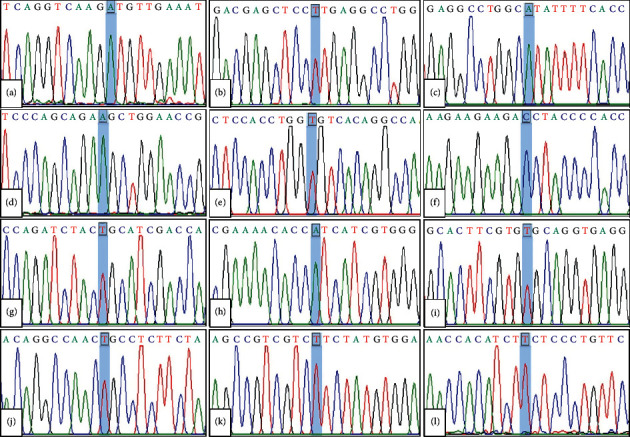
Electropherogram of 12 G6PD mutations identified in this study; (a) G6PD Viangchan (c.871G>A, p.Val291Met), (b) G6PD Canton (c.1376G>T, p.Arg459Leu), (c) G6PD Kaiping (c.1388G>A, p.Arg463His), (d) G6PD Mahidol (c.487G>A, p.Gly163Ser), (e) G6PD Quing Yan (c.392G>T, p.Gly131Val), (f) G6PD Aures (c.143T>C, p.Ile48Thr), (g) G6PD Coimbra (c.592C>T, p.Arg198Cys), (h) G6PD Songklanagarind (c.196T>A, p.Phe66Ile), (i) G6PD Union (c.1360C>T, p.Arg454Cys), (j) G6PD Valladolid (c.406C>T, p.Arg136Cys), (k) G6PD Chinese-5 (c.1024C>T, p.Leu342Phe), and (l) G6PD Mediterranean (c.563C>T, p.Ser188Phe).

Molecular analysis of the G6PD gene in G6PD-deficient Thai children identified a total of 12 different mutations (Table 2 & Figure 1). Among 104 G6PD alleles including 73 alleles from hemizygous males, 27 alleles from heterozygous females, and 4 alleles from homozygous/compound heterozygous females with G6PD deficiency, G6PD Viangchan (871G > A) was the most common mutation and was detected in 48 chromosomes (46.2%). G6PD Canton (1376G > T) was the second most common and was identified in 16 chromosomes (15.4%), followed by G6PD Kaiping (1388G > A) 15 chromosomes (14.4%) and G6PD Mahidol (487G > A) 9 chromosomes (8.6%). Eight uncommon G6PD mutations including 4 alleles (3.8%) of G6PD Quing Yan (392G > T), 2 alleles (1.9%) of G6PD Coimbra (592C > T), G6PD Union (1360C > T), G6PD Songklanagarind (196T > A), G6PD Valladolid (406C > T) and G6PD Aures (143C > T), and 1 allele (1%) of G6PD Chinese-5 (1024C > T) and G6PD Mediterranean (563C > T) were identified. G6PD Valladolid and G6PD Aures were firstly identified in Thai populations in our study. All 104 G6PD alleles were characterized by direct DNA sequencing. According to sex classification, all 12 mutations were detected among male patients, whereas only 6 mutations were identified among female patients potentially due to the low prevalence and smaller sample size of female patients (Table 2). Nevertheless, G6PD Viangchan was still the most common G6PD mutation identified among both male and female patients. Of the 29 female patients, the majority (27 patients, 93.1%) were identified to be heterozygous for G6PD mutations. Only 2 patients (6.9%) were identified to be either homozygous or compound heterozygous for G6PD mutations including 1 homozygous of G6PD Viangchan and 1 compound heterozygous of G6PD Viangchan and G6PD Canton.

Our study demonstrated the variability of G6PD levels ranging from very low detectable activities to some residual activity (Table 3). The average G6PD levels were 16.7°±°11.5 (3–76) IU/ml.RBC among affected males and 133.6°±°43.4 (9–195) IU/ml.RBC among heterozygous females. Two female patients who were homozygous and compound heterozygous for G6PD mutations had 35 and 10 IU/ml.RBC of G6PD level, respectively. G6PD levels, categorized by each type of G6PD mutations, are shown in Table 3. Interestingly, almost all affected males had G6PD levels less than 45 IU/ml.RBC except for one patient who was hemizygous for G6PD Valladolid presenting G6PD level 76 IU/ml.RBC. On the other hand, nearly all heterozygous females had G6PD levels more than 45 IU/ml.RBC, except for one individual (1 in 27 patients, 3.7%) who was heterozygous for G6PD Kaiping presenting G6PD level 9 IU/ml.RBC, which was in the affected male range.

Concerning genotype-phenotype correlation study, G6PD level was categorized by each clinical phenotype (Table 4). In G6PD-deficienct males, the average G6PD activities were 17.26°±°11.7 (3–76) IU/ml.RBC among neonatal jaundice patients and 11.8°±°8.9 (3–25) IU/ml.RBC among acute hemolytic patients. In G6PD-deficient heterozygous females, the average G6PD activities were 136.1°±°44.6 and 113.7°±°32.2 IU/ml.RBC among individuals presenting with neonatal jaundice and acute hemolytic anemia, respectively. Although G6PD levels among acute hemolytic individuals were slightly lower than those among neonatal jaundice individuals, no significant difference was found in the mean G6PD levels between these two major clinical phenotypes for both affected males (*p* value 0.135) and heterozygous females (*p* value 0.279).

## 4. Discussion

G6PD deficiency is seen primarily in populations across the Mediterranean, Africa, Middle East, and Southeast Asia where malaria was endemic, as the deficiency confers selective survival advantage from infection with *Plasmodium falciparum*. Consequently, the prevalence of G6PD deficiency is high, ranging from 5 to 20% in these regions [7]. In Thailand, the prevalence was 3 to 18% [1]. In this study, we aimed to characterize the genotype-phenotype correlation of the G6PD mutation in 102 unrelated Thai pediatric patients affected with G6PD deficiency including 73 males (71.6%) and 29 females (28.4%). As previously reported, the majority (89.2%) of our patients presented with neonatal hyperbilirubinemia followed by acute hemolytic anemia (10.8%) [5, 8]. Our study recruited patients with G6PD deficiency who were hospitalized and developed acute hemolysis or neonatal jaundice according to inclusion criteria described in the manuscript. This limitation might underestimate the actual prevalence of G6PD deficiency in Thailand due to overlooking those with mild symptoms or asymptomatic and could be the reason for the difference of prevalence between our study and the previous study [9, 10] that enrolled all patients who presented with intravascular hemolysis found 81% had G6PD deficiency at Gaza Strip, Palestine.

To date, more than 180 mutations are associated with G6PD deficiency [2, 3, 11]. Most mutations (85%) are missense variants which reduce G6PD enzyme stability and activity. In our study, 12 missense mutations affecting coding regions of the G6PD gene were identified. The three most common mutations were G6PD Viangchan (46.2%), G6PD Canton (15.4%), and G6PD Kaiping (14.4%). G6PD Viangchan has been previously reported to be the most common mutation in central (53.8%) and southern (31.3%) Thailand [5, 8]. In Southeast Asia, G6PD Viangchan is also the most common mutation identified in Cambodian (97.9%), Laotians (100%), Vietnamese (44%), and Malaysian Malay (37.2%) populations [12–15]. G6PD Mahidol which is the predominant variant in Myanmar (91.3%) and northern Thailand (20%) and also the common mutation among Malaysian Malays (15.1%) [15–17] was identified in 8.6% of G6PD-deficient patients in our study. The high prevalence of G6PD Viangchan and the existence of G6PD Mahidol among Thais probably suggested a common ancestral origin of the Thais and other Southeast Asian populations. G6PD Canton and G6PD Kaiping are the two commonest mutations among the Chinese [18] and were found to be the second (15.4%) and the third (14.4%) most common variants in our study, respectively. Other less common Chinese mutations including G6PD Quing Yan (3.8%), G6PD Union (1.9%), and G6PD Chinese-5 (1%) were also identified in smaller numbers. The discovery of these Chinese variants possibly indicated the descendants of Chinese immigrants in the Thai population. In addition, low frequency of three previously reported mutations among Thai G6PD-deficienct patients including G6PD Coimbra (1.9%), G6PD Songklanagarind (1.9%), and G6PD Mediterranean (1%) was also identified in this study.

Although novel G6PD mutation was not identified in our study, G6PD Aures and G6PD Valladolid were firstly identified among our Thai children affected with G6PD deficiency. G6PD Aures causing mild G6PD deficiency associated with favism was firstly discovered in Algeria in 1993 [19]. G6PD Valladolid was firstly identified in two unrelated Spanish males in 1997 [20]. This mutation causes mild hemolytic anemia. How these two mutations occurred among G6PD-deficient Thai children remains unclear. These mutations may arise independently, because no historical linkage was found between Spain, Algeria, and Thailand. Interestingly, both patients who carried the G6PD Valladolid variant live in northeastern Thailand. Because no study of G6PD variants has been conducted in northeastern Thailand, G6PD Valladolid may have a high prevalence in this geographic region.

Concerning X-linked inheritance, G6PD deficiency occurs more frequently among males than females as in our study for which the majority (71.6%) of patients comprised males (Table 1). Among hemizygous males and less common homozygous/compound heterozygous females, G6PD deficiency is fully expressed. Almost all affected males and two females who were either homozygous or compound heterozygous for G6PD mutations in our study exhibited G6PD levels less than 45 IU/ml.RBC. Only one affected male who carrying G6PD Valladolid had G6PD level 76 IU/ml.RBC suggesting a mildly affected G6PD variant [20]. Among heterozygous females, a combination of normal and G6PD-deficient cells could be identified due to X-inactivation in either one of the two X chromosomes during the early embryonic period making a wide range of G6PD enzymatic activity among carrier females [3]. In our study, the majority (93.1%) of female patients who had low G6PD levels was heterozygous for G6PD mutations. Nearly all heterozygous females had G6PD levels ranging from 61 to 195 IU/ml.RBC, which are higher than that in the affected male range. Only one heterozygous female (3.7%) carrying G6PD Kaiping had G6PD level 9 IU/ml.RBC, which is in the affected male range and possibly explained by nonrandom X-chromosome inactivation, which has been previously described in this type of mutation [21].

Concerning genotype-phenotype correlation, our data suggest no association between the genotypes, G6PD enzymatic activities, and clinical phenotypes among G6PD-deficient Thai children. The variability of G6PD activity is far from being explained by the type of mutation and is uncorrelated with clinical phenotypes including neonatal jaundice and acute hemolytic anemia which were the only two major clinical features identified in this study. Although G6PD levels remained poorly correlated with the genotype affected with neonatal jaundice and acute hemolytic anemia, it appeared to be better correlated with the genotype presented with chronic nonspherocytic hemolytic anemia (CNSHA) as described in related studies [22–24]. The lack of genotype-phenotype correlation in our study could be a consequence of a complex multifactorial mechanism probably related to both environmental factors and genetic modifiers such as infection, medications, and dietary pattern of G6PD-deficient individuals and the X-chromosome inactivation pattern among heterozygous [21].

Although various studies of G6PD mutation have been conducted in Thai populations, DNA sequencing of the G6PD gene was firstly used to comprehensively identify the mutation spectrum among Thai G6PD-deficient children. Polymerase chain reaction and restriction fragment length polymorphisms (PCR-RFLP) have been used to detect common G6PD mutations in Thai populations in central, southern, and northern Thailand [5, 8, 17]. The mutations were unidentified in approximately 9 to 23% of subjects suggesting a greater genetic heterogeneity than previously anticipated. Our study demonstrated the efficiency of Sanger sequencing which could identify mutations among all 102 patients. In contrast to PCR-RFLP assays which are limited to detecting only known variants, direct sequencing is more comprehensive and reliable for detecting sequence variations within the genes.

## 5. Conclusion

In conclusion, this study characterizes the molecular heterogeneity of G6PD variants causing G6PD deficiency among Thai children. All of the G6PD mutations have been characterized by direct DNA sequencing of G6PD genes. Twelve different G6PD mutations were identified in our study. G6PD Viangchan, G6PD Kaiping, and G6PD Canton were the three most common mutations identified in our study and accounted for more than 75% of our patients. Although genotype-phenotype correlation was not demonstrated in our study, molecular analysis of the G6PD gene remains useful for diagnostic confirmation and carrier detection leading to appropriate genetic counseling for patients and their family members in the future.

## Figures and Tables

**Table 1:** Clinical phenotypes of 102 G6PD-deficient Thai children.

Clinical phenotype (*n*)	Male *n* (%)	Female *n* (%)	Total (102)
Neonatal jaundice (91)	66 (90.4)	25 (86.2)	91 (89.2)
Acute hemolytic anemia (11)	7 (9.6)	4 (13.8)	11 (10.8)
Total (102)	73 (71.6)	29 (28.4)	102

Data shown as number (percent).

**Table 2:** The genotyping of 104 G6PD alleles in 102 G6PD-deficient Thai children.

Genotype of G6PD mutation	Number *n* (%)	Male	Female	
		Hemizygous *n* (%)	Heterozygous *n* (%)	Homozygous/compound heterozygous *n* (%)
Viangchan (871G > A)	48 (46.2)	34 (46.6)	11 (40.7)	3 (75%)
Canton (1376G > T)	16 (15.4)	9 (12.3)	6 (22.2)	1 (25%)
Kaiping (1388G > A)	15 (14.4)	12 (16.4)	3 (11.1)	—
Mahidol (487G > A)	9 (8.6)	6 (8.2)	3 (11.1)	—
Quing Yan (392G > T)	4 (3.8)	1 (1.3)	3 (11.1)	—
Aures (143C > T)	2 (1.9)	2 (2.7)	—	—
Coimbra (592C > T)	2 (1.9)	2 (2.7)	—	—
Songklanagarind (196T > A)	2 (1.9)	2 (2.7)	—	—
Union (1360C > T)	2 (1.9)	2 (2.7)	—	—
Valladolid (406C > T)	2 (1.9)	1 (1.3)	1 (3.7)	—
Chinese-5 (1024C > T)	1 (1)	1 (1.3)	—	—
Mediterranean (563C > T)	1 (1)	1 (1.3)	—	—
Total	104	73	27	4

Data shown as number (percent).

**Table 3:** Genotype of G6PD mutations and G6PD levels in 73 affected males and 27 heterozygous females.

Genotype of G6PD mutation (*n*)	G6PD level (IU/ml.RBC) (mean±SD (min–max))	
	Affected males	Heterozygous females
Viangchan (45)	17.18 ± 9.35 (5–45)	138.63 ± 34.2 (61–189)
Canton (15)	12.6 ± 7.72 (3–26)	137.17 ± 53.04 (84–195)
Kaiping (15)	17.92 ± 10.91 (7–36)	9, 129, 180
Mahidol (9)	15.5 ± 7.77 (8–29)	108, 109, 136
Quing Yan (4)	13	100, 152, 177
Aures (2)	3, 10	—
Coimbra (2)	23, 34	—
Songklanagarind (2)	3, 28	—
Union (2)	9, 10	—
Valladolid (2)	76	159
Chinese-5 (1)	25	—
Mediterranean (1)	15	
Total (100)	16.7 ± 11.5 (3–46)	133.6 ± 43.4 (9–195)
	(73)	(27)

Data shown as mean±SD or number (percent).

**Table 4:** G6PD levels categorized by the genotype of G6PD mutations and clinical phenotypes of 73 affected males and 27 heterozygous females.

Genotype of G6PD mutation	G6PD level categorized by clinical phenotype (IU/ml.RBC) (mean±SD (min–max))			
	Neonatal Jaundice		Acute hemolytic anemia	
	Affected male (66)	Heterozygous female (24)	Affected male (7)	Heterozygous female (3)
Viangchan	16.8 ± 9.6 (5–45)	125.2 ± 53.7 (61–189)	8	148
Canton	12.3 ± 8.2 (3–26)	123.7 ± 76 (85–195)	3	84
Kaiping	11.8 ± 11.3 (7–34)	9, 129, 180	12, 25	-
Mahidol	11.75 ± 4.5 (8–17)	108, 136	—	109
Quing Yan	13	100, 152, 177	—	—
Aures	10	—	3	—
Coimbra	34	—	23	—
Songklanagarind	3, 28	—	—	—
Union	10	—	9	—
Valladolid	76	159	—	—
Chinese-5	25	—	—	—
Mediterranean	15	—	—	—
G6PD level (IU/ml.RBC) (mean±SD (min–max))	17.26 ± 11.7 (3–76)	136.1 ± 44.6 (9–195)	11.8 ± 8.9 (3–25)	113.7 ± 32.2 (84–148)

Data shown as mean+_SD or number (percent).

## Data Availability

The data used to support the findings of this study are included within the article and available from the corresponding author upon request.
